# Anti-inflammatory and analgesic effects of *Streblus indicus*

**DOI:** 10.3389/fphar.2023.1249234

**Published:** 2023-09-27

**Authors:** Yan-Qing Xie, Jing-Yao Huang, Yun-Xiu Chen, Qian Zhou, Qi-Xiu Zhou, Zhu-Ya Yang, Shi-Kui Xu, Wen-Hong Tan, Lu Liu

**Affiliations:** ^1^ Yunnan Yunzhong Institute of Nutrition and Health, Yunnan University of Chinese Medicine, Kunming, China; ^2^ Yunnan Institute for Food and Drug Control, Kunming, China

**Keywords:** *Streblus indicus* (Bur.) Corner, anti-inflammation, analgesia, nuclear factor kappa B, inflammatory cytokines

## Abstract

The bark of *Streblus indicus*, a Dai medicine in China, has been listed in the Chinese Materia Medica as possessing hemostatic and analgesic properties. Ethnic medicine books record that its bark or leaves for the treatment of mumps and lymphoma. However, according to the literature survey, anti-inflammatory and analgesic studies available for leaves and branches of *S. indicus* have been seldom reported so far. The current study focuses on the metabolites of *S. indicus* bark and leaves responsible for anti-inflammatory and analgesic effects on the basis of bioactive-included acetic acid writhing, hot-plate, and xylene-induced ear swelling. The secretion of inflammatory mediators, TNF-*α*, IL-6, IL-1*β*, IL-4, and IL-10, were evaluated for their anti-inflammatory by xylene-induced in mouse ear cells. Histological examination was used to assess the anti-inflammatory and analgesic effects of the branches and leaves of *S. indicus*, and Western blot analysis determined the mechanism of the methanolic extract of branches and leaves. Different metabolites of *S. indicus* significantly alleviated analgesic and anti-inflammatory effects, with no discernable differences among them. All metabolites decreased the levels of TNF-*α*, IL-1*β*, and IL-6 and increased the levels of IL-4 and IL-10. The analgesic and anti-inflammatory mechanism of the methanolic extract was related to the NF-*k*B signaling pathway. These results not only would account for scientific knowledge for the traditional application of *S. indicus*, but also provide a credible theoretical foundation for the further development of anti-inflammatory and analgesic agents.

## 1 Introduction

Inflammation, a defensive response, typically manifests as redness, swelling, heat, pain, and dysfunction at the site of infection or trauma (Shaik-Dasthagirisaheb et al., 2013). It is an integrated process of damage and anti-damage (Elkahloun et al., 2019; De Meij et al., 2021). In the process of inflammation, damaging factors directly or indirectly cause tissue and cell damage; the damaged tissues or cells dilute and kill damaging factors through inflammatory congestion and exudation; simultaneously, damaged tissue can be repaired and healed through the regeneration of parenchymal and interstitial cells (Hu et al., 2020; Zhang et al., 2021; Gugliandolo et al., 2021; Maria, 2021; Sophie et al., 2022). Inflammation can manifest as either acute or chronic. Acute processes are transient and usually recover, while chronic processes arise from the transformation of inflammatory processes that are too strong or do not reach the stage of resolution (Arulselvan et al., 2016). Chronic inflammation may be harmful and known to cause other inflammation-related diseases, such as atherosclerosis and systemic lupus erythematosus (Ballester et al., 2022; Henein et al., 2022). Excessive inflammation may result in a life-threatening condition. Nonsteroidal anti-inflammatory drugs (NSAIDs), such as aspirin and diclofenac sodium, are efficacious in treating inflammation-related diseases, rendering them the preferred medications for analgesia and anti-inflammation. However, these drugs and other synthetic preparations have been associated with many acute and chronic side effects, including increased risk of heart disease with long-term use (Alam et al., 2022). That has led to a search for alternative therapies with minimal or no side effects to treat pain and inflammation.

*Streblus indicus* (Bur.) Corner is a traditional medicine used by the Dai people in Yunnan for hemostasis and pain relief. Its distribution includes China, Malaysia, India, and Thailand. The bark of the plant could reduce swelling and alleviate pain (Yunnan Food and Drug Administration, 2013). Meanwhile, according to records from ethnic medicine books, the bark or leaves could alleviate mumps and submandibular lymphoma pain (Lin et al., 2003). Modern pharmacological studies have shown that the coumarin compounds from the bark metabolite of *S*. *indicus* have potent anti-inflammatory and analgesic effects (Zhang et al., 2021). Although *S*. *indicus* has traditional use for treating inflammatory diseases, there are only a few reports on the biological activities of its branches and leaves, requiring further studies.

In this paper, we conducted three classical experiments on analgesic and anti-inflammatory effects to evaluate and compare the activities of the bark, branches and leaves of *S*. *indicus*. Furthermore, we measured TNF-*α*, IL-1*β*, IL-6, IL-4, and IL-10 levels, and investigated the anti-inflammatory activity of the branches and leaves by histological analysis. We also explored the effect of the plant on the NF-*κ*B pathway. This study offers scientific evidence to explain the traditional medicinal use of *S*. *indicus*.

## 2 Materials and methods

### 2.1 Plants and materials

The bark, branches and leaves of *S. indicus*, a traditional herbal medicine with the Chinese name Hua Ye Die Da, were collected from Menla County, Xishuangbanna City, Yunnan Province in May 2019, and verified by Professor Wenhong Tan. The voucher specimens (number: SI20190510) were deposited at the Engineering Research Center of Chinese Herbal Pieces at the University of Yunnan Province (Yunnan University of Chinese Medicine).

Diclofenac sodium was purchased from Shanghai Macklin Biochemical Technology Co., Ltd. (Shanghai, China). Aspirin and radioimmunoprecipitation assay (RIPA) lysis buffer were purchased from Beijing Solarbio Science and Technology Co., Ltd. (Beijing, China). Sodium carboxymethyl cellulose (CMC-Na) was purchased from Sinopharm Chemical Reagent Co., Ltd. (Shanghai, China). ELISA kits (96T) for tumor necrosis factor *α* (TNF-*α*), interleukin 4 (IL-4), interleukin 1*β* (IL-1*β*), interleukin 10 (IL-10) and interleukin 6 (IL-6) were obtained from Jiangsu Meimian Industrial Co., Ltd. (Jiangsu, China). The antibody to nuclear factor-kappa-B (NF-*κ*B), inhibitor-kappa-B alpha (IkB*α*), phospho-nuclear factor-kappa-B (*p*-NF-*κ*B), phospho-inhibitor-kappa-B alpha (*p*-IkB*α*) were obtained from Beijing Biosynthesis Biotechnology Co., Ltd. (Beijing, China). Beta-actin (β-actin) antibody and horseradish peroxidase (HRP) rabbit anti-goat were provided by Proteintech Group, Inc. (Wuhan, China). Bicinchoninic acid (BCA) protein assay kit and SDS-PAGE gel configuration kit were provided by Beyotime Biotech Inc. (Shanghai, China). Polyvinylidene difluoride (PVDF) membrane was provided by Millipore. Ice acetic acid, xylene, and anhydrous ethanol were analytical reagents. Ultrapure water was used as a solvent in the model group and administration groups, and 0.5% sodium carboxymethyl cellulose was used as a suspension aid.

### 2.2 Animals

The SPF Kunming (KM) mice, 150 female mice and 150 male mice (18–22 g), were provided by Kunming Chushang Technology Co., Ltd. (Kunming, China) [License No. SCXK (Hunan) 2019-0004]. Mice were housed in a standard laboratory at 24°C ± 3°C with a 12-h dark/light cycle and provided with *ad libitum* water and food for 7 days. The mice were randomly assigned to model group (M), positive drug group (P), low dose group of aqueous extract of bark (LAB, 5.77 mg/kg), high dose group of aqueous extract of bark (HAB, 23.08 mg/kg), low dose group of aqueous extract of branches and leaves (LABL, 5.77 mg/kg), high dose group of aqueous extract of branches and leaves (HABL, 23.08 mg/kg), low dose group of methanolic extract of bark (LMB, 46.16 mg/kg), high dose group of methanolic extract of bark (HMB, 184.64 mg/kg), low dose group of methanolic extract of branches and leaves (LMBL, 46.16 mg/kg), high dose group of methanolic extract of branches and leaves (HMBL, 184.64 mg/kg) in the following experiments. The entire animal experiment was conducted in accordance with the internationally accepted guidelines for the use and care of laboratory animals, and the guidelines of the Animal Welfare Law of China. The detailed experimental procedures involving animals were approved by the Laboratory Animal Ethics Committee of the Yunnan University of Chinese Medicine (protocol code SYXK (DIAN) K2017-0005).

### 2.3 Cell culture

Mouse mononuclear macrophage leukemia cells (RAW 264.7) were cultured in a culture plate containing 5 mL DMEM complete medium (90% DMEM basal medium +10% FBS), and the culture plate was placed in an incubator (temperature 37°C + CO_2_ 5%) until the cell growth state was stable and moderate (Duan et al., 2021; Xue et al., 2021). The experimental groups were set up in advance as the normal control group (only complete medium > DMEM) and drug administration group (concentrations of 25, 50, 100, 200, and 300 μg/mL). For each experimental trial, wells were transfected in triplicate and each well was assayed in triplicate.

### 2.4 Sample preparation

Equal amounts of the bark, branches and leaves of *S. indicus* were weighed, crushed, and extracted twice with 95% methanol at 52°C for 1 h each time. The residues and filtrates of bark, branches and leaves of *S. indicus* were obtained through filtration. The filtrates were lyophilized to obtain the methanolic metabolites of bark, branches and leaves of *S. indicus*, with an extraction rate of about 8%. The residues of bark, branches and leaves were extracted twice with water at 80°C, then precipitated with 80% ethanol, and the precipitated parts were lyophilized to obtain the aqueous metabolites of bark, branches and leaves of *S. indicus*, with an extraction rate of about 5%.

### 2.5 Animal testing

#### 2.5.1 Acetic acid writhing test

Four-week-old Kunming mice (18–22 g), half male and half female, were randomly divided into ten groups, with ten mice in each group. Mice in the positive control group were treated with aspirin (150 mg/kg) by gavage, while the other groups were treated with a corresponding metabolite for 5 days. On the last day, 0.6% acetic acid was intraperitoneally injected 1 h after administration. The number of writhing reactions of each mouse within 15 min after acetic acid injection was recorded, and the data were summarized to make graphs (Han and Song, 2008; Wang et al., 2020).

#### 2.5.2 Hot-plate test

A total of 100 female KM mice, weighing 18–22 g and exhibiting pain durations ranging from 10–30 s, were selected before the experiment. Before dosing, each mouse was placed on a heated plate (55°C ± 0.5°C) twice, with a 15-min interval between placements, to determine the average pain threshold. Based on the initial pain threshold determination, the mice were divided into groups of 10 for the experiment. After adaptive feeding, the mice in the positive control group received diclofenac sodium (10 mg/kg) by gavage, and the mice in the remaining groups were given the corresponding metabolite by intragastric administration for 7 days. Pain thresholds were measured at 30, 60, 90, 120, and 150 min after the last administration using an intelligent hot-plate instrument, and the rate of increase in pain threshold was obtained. A maximum of 20 s was set as the cut-off time to avoid tissue damage (Garateix et al., 2011; Arya et al., 2016; Huang et al., 2021). The calculation formula was as follows:

$$\text{Percentage }\left(\%\right)=\frac{\text{Mean}\;\text{threshold}\;\text{of}\;\text{pain}\;\left(\text{after}\right)-\text{Mean}\;\text{thershold}\;\text{of}\;\text{pain}\;\left(\text{before}\right)}{\text{Mean}\;\text{threshold}\;\text{of}\;\text{pain}\;\left(\text{before}\right)}\times 100\%$$

#### 2.5.3 Xylene-induced ear swelling test

One hundred male KM mice weighing 18–22 g each were randomly split into groups of ten mice. After adaptive feeding, the mice in the positive control group were intragastrically administered diclofenac sodium (10 mg/kg), and the mice in the other groups were intragastrically administered the corresponding metabolite for 1 week. 30 min after the final administration, 20 µL of xylene was evenly applied on both sides of the right ear, the left ear was considered as the control. Mice were then sacrificed by cervical dislocation and the ears were removed along the auricular baseline 40 min later. An 8 mm diameter circular punch was used to punch holes in the same place in the left and right ears of the same mice to obtain equally sized ear slices. The difference in weight between the left and right ears of the same mouse was immediately calculated and recorded, i.e., the degree of swelling (Agrawal et al., 2015; Jain et al., 2016; Li et al., 2020). Inflammatory inhibition rate (IR) was defined as the difference between the degree of swelling in the control and treatment groups and the ratio of degree of swelling in the control group.

$$\text{IR}\%=\frac{\text{Mean}\;\text{ear}\;\text{edema}\;\text{rate}\;\left(\text{control}\right)-\text{Mean}\;\text{ear}\;\text{edema}\;\text{rate}\;\left(\text{test}\right)}{\text{Mean}\;\text{ear}\;\text{edema}\;\text{rate}\;\left(\text{control}\right)}\times 100\%$$

After the swelling test, the right ears of each group and the lift ears of the model group were placed in EP tubes, frozen in liquid nitrogen, and stored in a refrigerator at −80°C until use. For histological examination, the left and right ears of mice from the xylene-induced edema model and the right ear of the HMBL group were sectioned, dehydrated, stained with eosin, counterstained with hematoxylin, dehydrated again, cleared, and sealed. The staining effect was observed under a microscope and photographed for subsequent analysis (Dong et al., 2017; Ramírez et al., 2022).

#### 2.5.4 Inflammation factors tests

The right earpiece of each administration group obtained from the xylene swelling experiment in the EP tube was thawed, placed in phosphate-buffered saline (PBS, PH 7.4) at 2–8°C, and processed using a tissue homogenizer. The supernatant was collected by centrifugation, 2000–3,000 rpm for 20 min. Then, according to the ELISA kit instructions (Chen et al., 2020), the levels of three pro-inflammatory and two anti-inflammatory factors, TNF-*α*, IL-4, IL-1*β*, IL-10, and IL-6, were determined. Samples were amplified at the ratio of 10 mg sample + 100 μL PBS and the standard curve was plotted. The OD value of the sample was substituted to calculate the sample concentration, and the actual sample concentration was calculated by multiplying the dilution multiple (Li et al., 2020).

#### 2.5.5 Western blot analysis of anti-inflammatory NF-*κ*B pathway in ear swelling test

For Western blot analysis, we used the left (NC) and right (M) ears of mice from the xylene-induced edema model along with the right ear of the HMBL group to investigate the anti-inflammatory protein pathway. The reagents were prepared following the manufacturer’s instructions. Frozen samples were homogenized in RIPA buffer supplemented with protease inhibitors. The supernatant was obtained by centrifugation, at 10,000 rpm for 10 min. We determined the protein concentration using a BCA assay. Protein from each sample was separated by 10% sodium SDS-PAGE and transferred to PVDF membranes. The membrane was blocked with 5% nonfat milk for 2 h followed by incubation with specific antibodies NF-*κ*B/*p*65, *p*-NF-*κ*B/*p*65, IkB*α*, and *p*-IkB*α* overnight at 4°C. After washing with TBST, the membranes were incubated with the secondary antibodies. ECL detection reagent was applied to detect the membrane signal. The bands were quantified using Image J, and the results were analyzed using Excel and GraphPad Prism (Zhuang et al., 2017; Jiang et al., 2019; Mahmoud et al., 2019).

### 2.6 Cytotoxicity assay

RAW 264.7 cells were inoculated into 96-well plates at 1 × 10^5^/mL, and 100 μL of cell suspension was inoculated into each well, cultured for 24 h, and the supernatant discarded (Murakami et al., 2018). Different concentrations of extracts were added to the wells, with four duplicate wells set up for each concentration. Cells were incubated at 37°C and 0.5% CO_2_ concentration for 24 h after drug addition. The 96-well plate was removed, and the cell supernatant was discarded. Next, 100 μL of CCK8 (complete medium: CCK8 = 10:1) was added to the plate. The OD values for each group were measured and recorded under a microplate reader at 450 nm wavelength, and then the cell viability plot of each drug was obtained (Zeng et al., 2016).

### 2.7 Statistical analysis

Data was analyzed using GraphPad Prism software (8.4.0.671) and presented as mean ± SD. Data were evaluated by multiple comparisons in one-way ANOVA comparing the mean of each column and control column, combined with a T-test. Comparisons that yielded *p*-values less than 0.05 were deemed statistically significant (*p* < 0.05).

## 3 Results

### 3.1 Acetic acid writhing test

**FIGURE 1:**
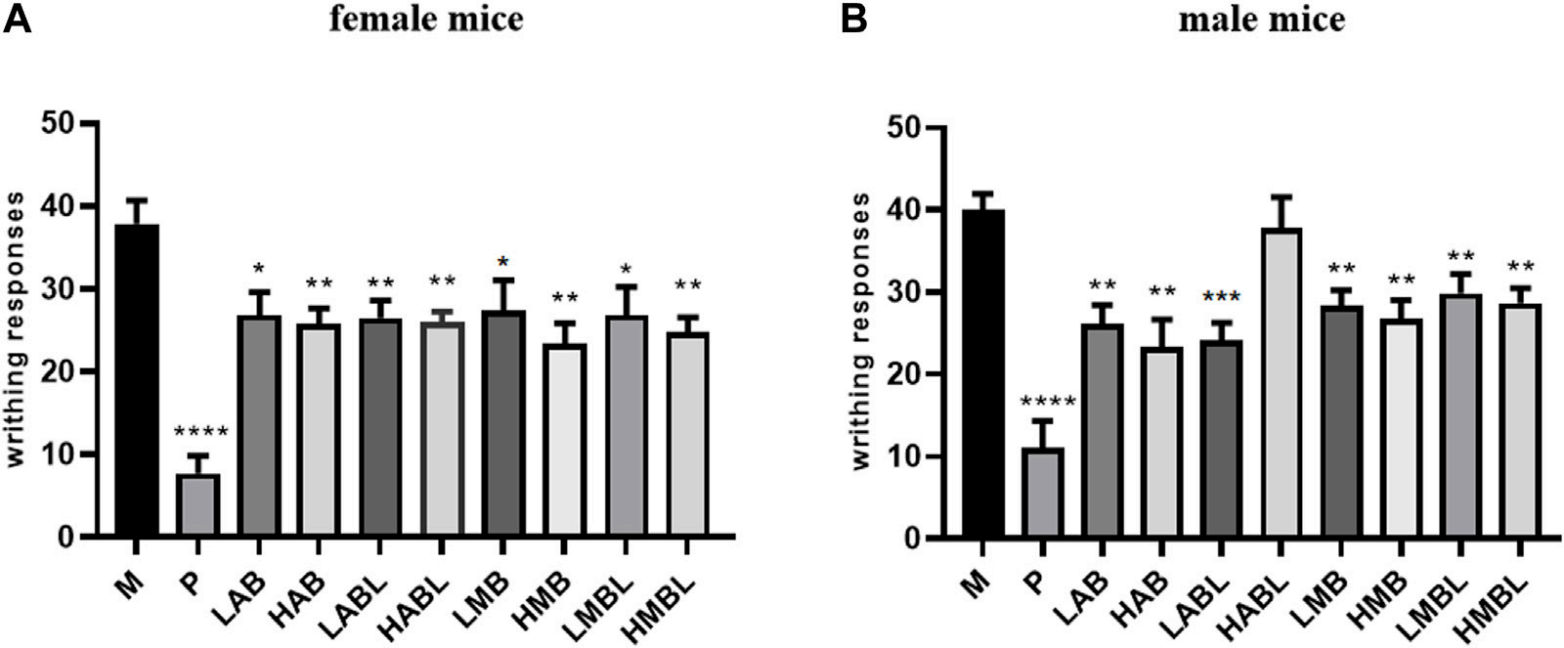
The results of acetic acid writhing test. **(A)** Number of turns in female mice. **(B)** Number of turns in male mice. *p < 0.05, **p < 0.01, ***p < 0.001, ****p < 0.0001 compared with model group.

As shown in Figure 1, the metabolites of bark, branches and leaves reduced the number of writhing responses compared to the M group, indicating that they have significant analgesic effects on chemical stimulation-induced pain induced by acetic acid (*p* < 0.05). Aside from LABL and HABL groups of male mice, all administered groups exhibited a dose-dependent trend in their analgesic effects. However, the analgesic potency of all metabolites is inferior to that of the P group. In addition, a comparison was made between the analgesic effects of the bark, branches and leaves. Results showed that except for the HAB and HABL groups in male mice, the bark, branches and leaves of *S. indicus* were similar in reducing the number of 0.6% acetic acid-induced writhing responses (*p* > 0.05), indicating that they had similar effects on analgesia.

### 3.2 Hot-plate test

As shown in Table 1, compared with the M group, the rate of increase in the pain threshold of each experimental group at different time periods after administration was significantly increased, indicating that the analgesic effect was significant, and continued up to 150 min after administration (*p* < 0.05). Nonetheless, the efficacy of all metabolite treatments was less prominent than that of the *P* group. The study compared the analgesic effects of different extraction methods and different extraction parts. The findings indicated that the aqueous and methanolic extracts from bark or branches and leaves showed similar rates of increase in pain threshold at each time point after administration. In addition, the difference in the rate of increase in pain threshold between the branches and leaves and the bark was minimal, indicating that they had similar analgesic effects.

**TABLE 1:** The Results of Hot-plate Pain (‾x±s, *n* = 10).

Groups	Dose (mg/kg)	Initial average pain threshold/s	Pain threshold at different time after administration (s)					The increase rate of pain threshold at different time (%)				
			30 min	60 min	90 min	120 min	150 min	30 min	60 min	90 min	120 min	150 min
M	-	16.93 ± 6.56	14.20	12.97	11.86	13.54	16.3	−16.13	−23.39	−29.95	−20.02	−3.72
P	10.00	16.67 ± 6.85	22.74	23.26	26.56	33.47	33.81	36.41	39.53	59.33	100.78	102.82
LAB	5.77	18.54 ± 6.99	16.43	17.89	18.80	22.84	22.82	−11.38	−3.51	1.40	23.19	23.09
HAB	23.08	16.87 ± 3.92	18.76	19.47	21.66	25.35	26.89	11.20	15.41	28.39	50.27	59.40
LABL	5.77	16.44 ± 5.99	16.99	18.71	18.50	22.94	22.90	3.35	13.81	12.53	39.54	39.29
HABL	23.08	16.45 ± 4.86	18.22	21.58	21.82	25.99	28.58	10.76	31.19	32.64	57.99	73.74
LMB	46.16	15.94 ± 4.44	16.73	18.98	19.13	22.91	25.32	4.96	19.07	20.01	43.73	58.85
HMB	184.64	17.95 ± 5.23	18.81	20.66	22.34	26.09	29.19	4.79	15.10	24.46	45.35	62.62
LMBL	46.16	14.21 ± 3.49	16.65	18.28	19.91	23.07	26.12	2.71	12.77	22.83	42.32	61.14
HMBL	184.64	17.85 ± 6.04	18.97	22.59	23.71	29.62	29.03	6.27	26.55	32.83	65.94	62.63

### 3.3 Xylene-induced ear swelling test

**FIGURE 2:**
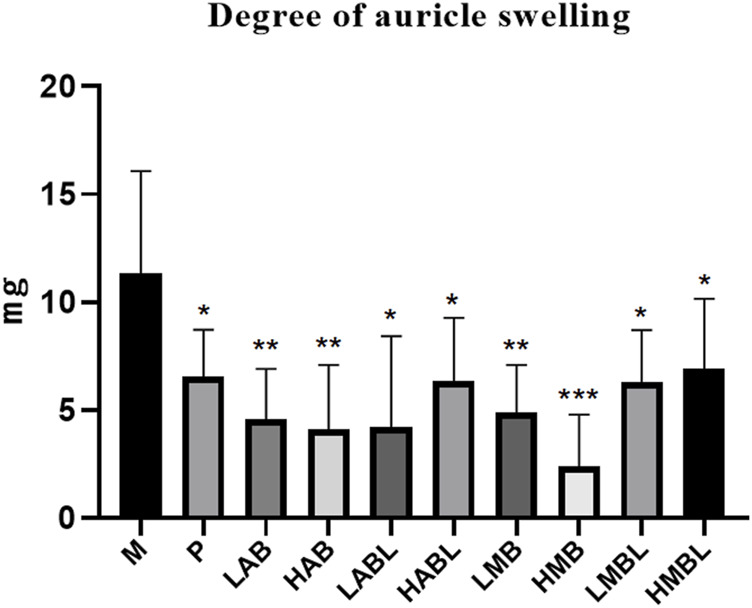
The results of xylene-induced ear swelling test. *p < 0.05, **p < 0.01, ***p < 0.001 compared with model group.

The results of the xylene swelling test results are displayed in Figure 2. Compared to the M group, the aqueous and methanolic metabolites of the bark, branches and leaves of *S. indicus* exhibited significantly increased inflammation inhibition rates (*p* < 0.05), demonstrating significant anti-inflammatory effects. The HMB group (184.64 mg/kg body weight) reduced the degree of ear swelling, which was better than the *P* group (*p* < 0.05). Comparison of the experiment results using methanolic and aqueous extracts from bark, branches and leaves revealed that extracts from any part of *S. indicus*, whether aqueous or methanolic, inhibited ear swelling to a similar extent. Ear swelling inhibition was comparable for bark and branches extracted using the same solvent, except for the HMBL and HMB groups.

### 3.4 Inflammatory factor assay

**FIGURE 3:**
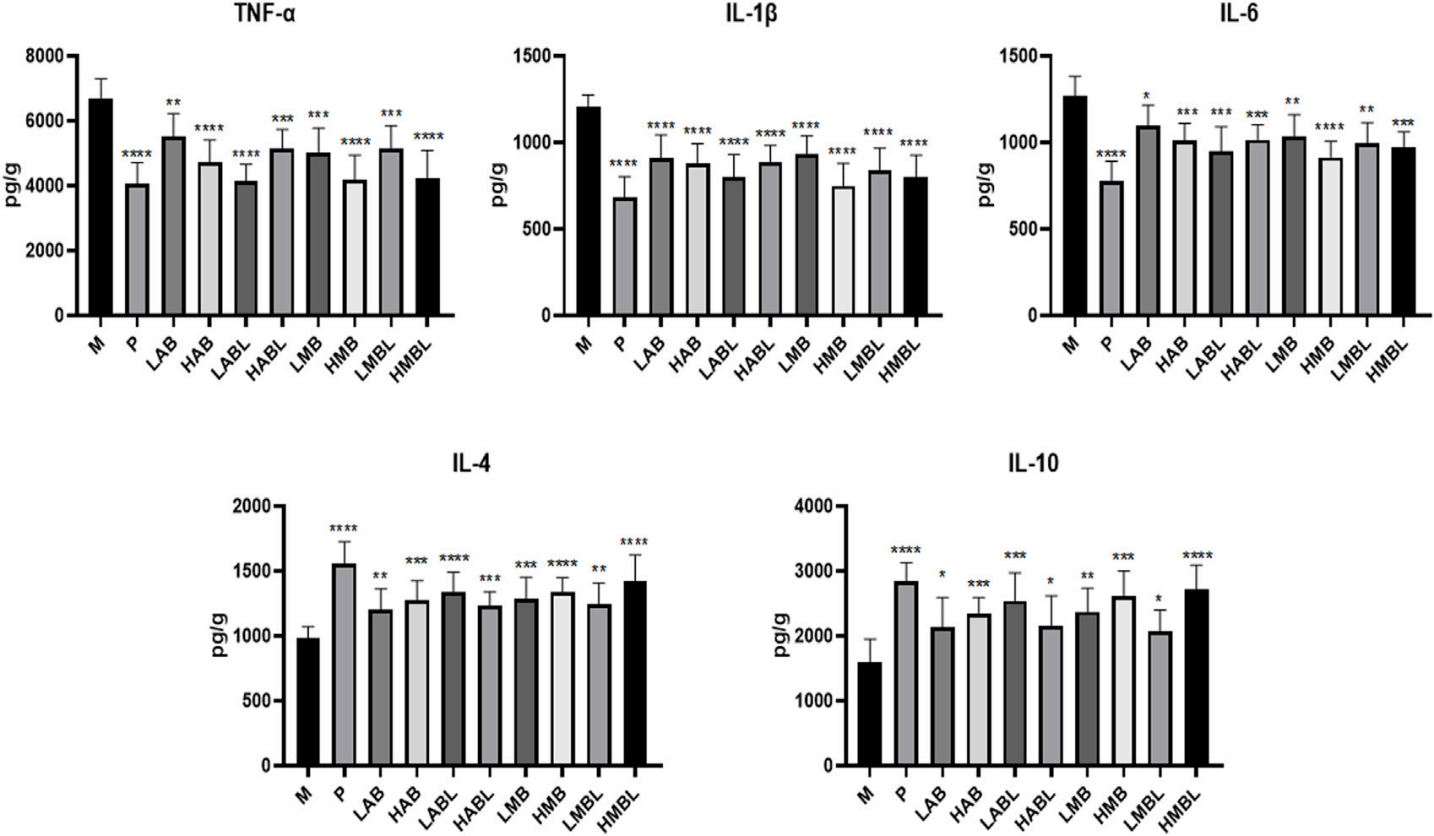
The results of inflammatory cytokines assay. *p < 0.05, **p < 0.01, ***p < 0.001, ****p < 0.0001 compared with model group.

The results of the inflammatory cytokine assay are shown in Figure 3. Compared with the M group, the metabolites from the bark, branches and leaves of *S. indicus* significantly inhibited TNF-*α*, IL-1*β*, and IL-6 levels, and promoted IL-4 and IL-10 levels (*p* < 0.05). With the exception of the LABL and HABL groups, all administration groups exhibited dose-dependent effects on the levels of TNF-*α*, IL-1*β*, and IL-6, as well as up-regulatory effects on the levels of IL-4 and IL-10. It is worth noting that the inhibition of TNF-*α* in the LABL, HMB, and HMBL groups was nearly equivalent to that in the P group. The effects of different extraction methods and different extraction parts on the levels of TNF-*α*, IL-1*β*, IL-6, IL-4, and IL-10 were compared. The results showed that the pharmacological effects of aqueous and methanolic metabolites from the same extraction part on these five inflammatory factors were similar. The variances in the inhibition of TNF-*α*, IL-1*β*, and IL-6 levels and promotion of IL-4 and IL-10 levels were minimal between bark and branches and leaves extracted using the identical method of extraction, except for LAB and LABL groups. These results indicated that the metabolites of bark, branches and leaves of *S. indicus* had significant anti-inflammatory effects, which were related to the reduction of TNF-*α*, IL-1*β*, IL-6, and promotion of IL-4 and IL-10 levels.

### 3.5 Histological examination analysis

**FIGURE 4:**
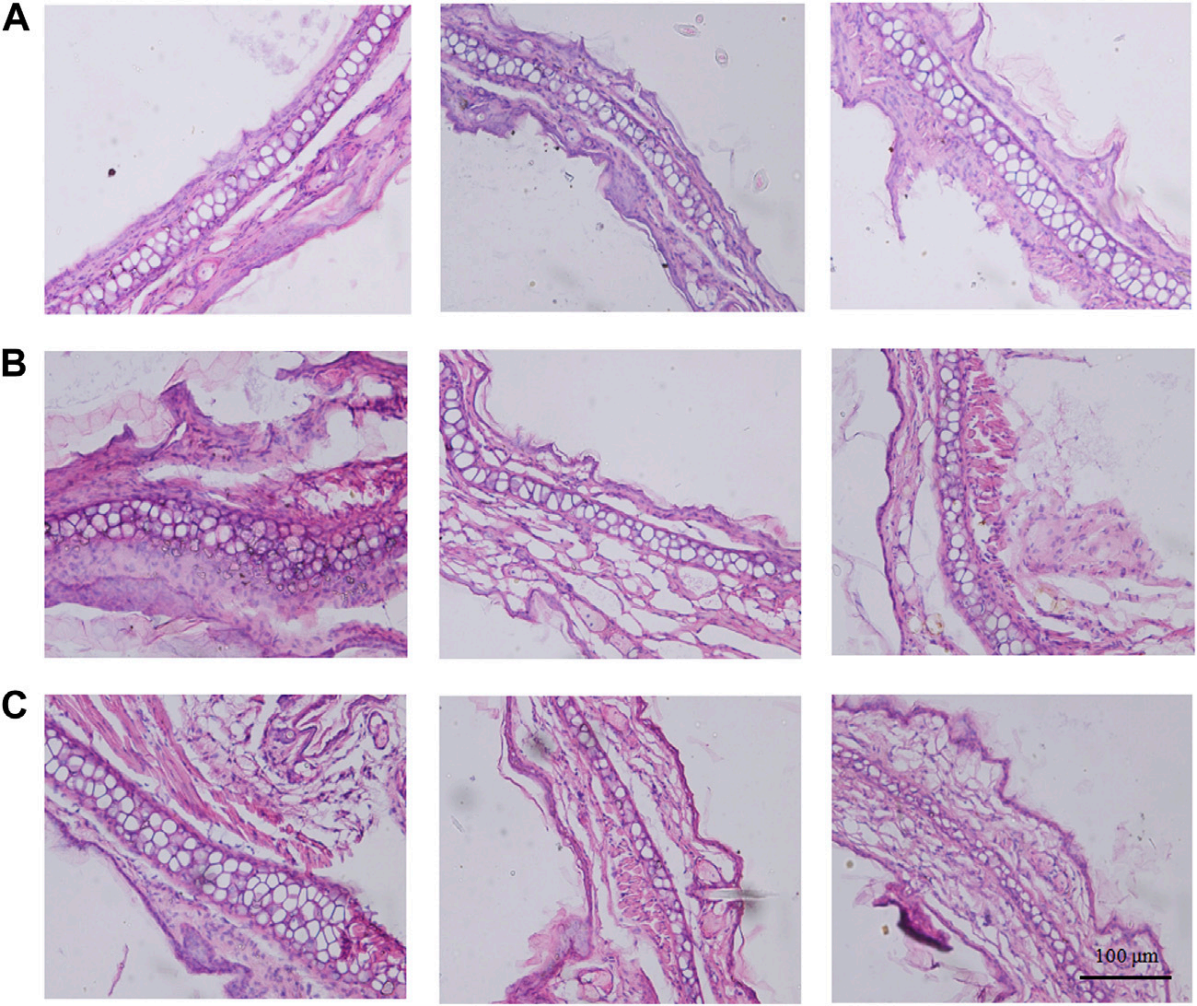
The results of histological examination analysis. **(A)** Histological alteration of the left ear in model group mice. **(B)** Histological alteration of the right ear in model group mice. **(C)** Histological alteration of the right ears from high dose group of methanolic extract of branches and leaves.

To objectively evaluate the potential anti-inflammatory impact of the branches and leaves of *S. indicus*, the histological examination analysis considered the right ears of the HMBL group with the best anti-inflammatory effect in the prior experiment, as well as both ears of the M group. As shown in Figure 4, the left ear featured neatly arranged inner and outer membranes (Figure 4A), whereas the right ear showed a thickening and disordered membrane structure with clustered nuclei (Figure 4B). Compared with the M group, the HMBL group reduced the thickness of the outer ear membrane in mice and restored the arrangement of certain membrane structures (Figure 4C).

### 3.6 Results of anti-inflammatory NF-*κ*B pathway in ear swelling test

**FIGURE 5:**
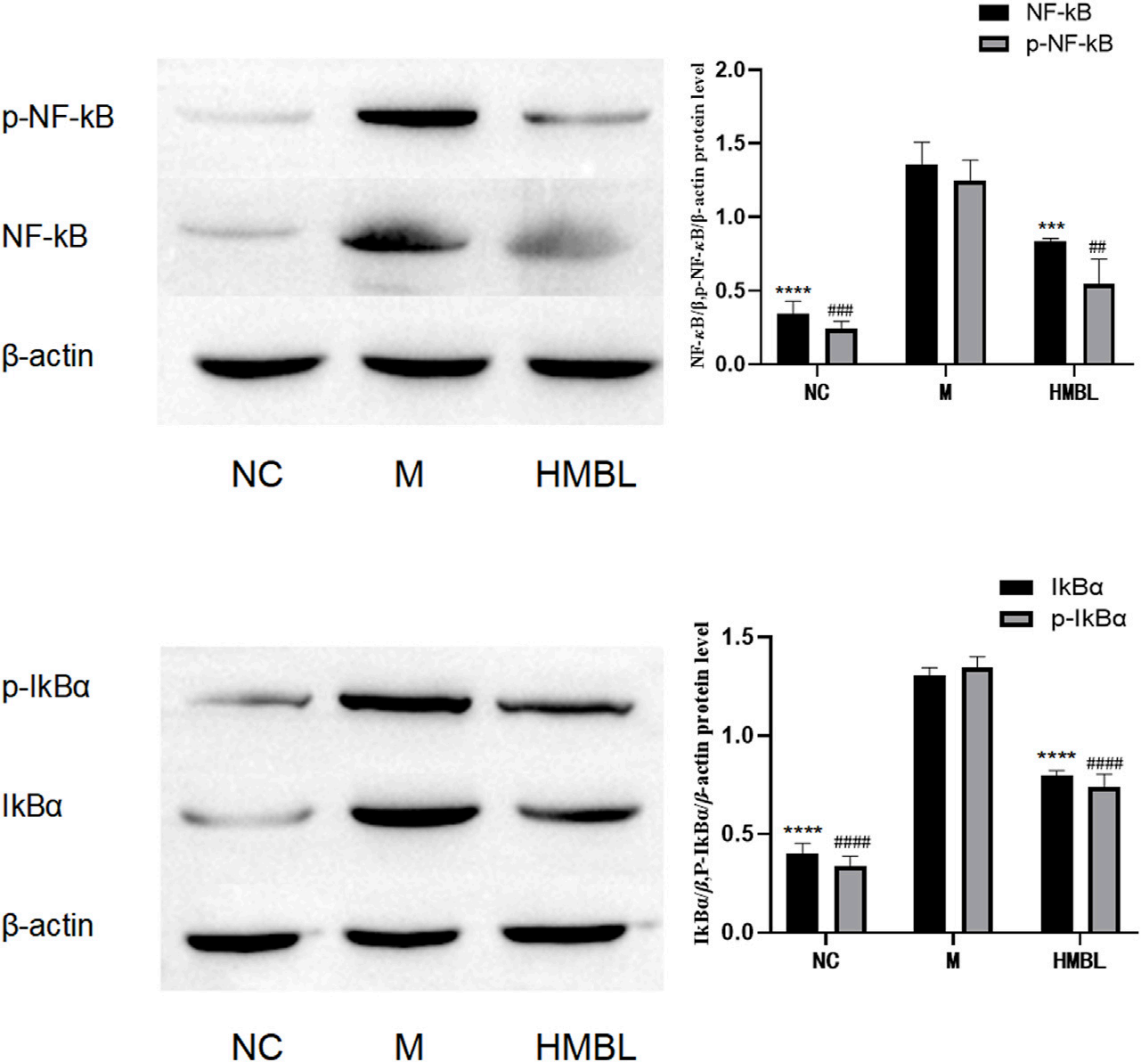
The results of NF-*κ*B pathway in ear swelling anti-inflammatory test. The experimental groups were divided into blank control group (NC), model group (M) and high dose group of methanolic extract of branches and leaves (HMBL). In the figure, * is used to represent *p* values for NF-*κ*B and IkBα groups, and # is used to represent *p* values for *p*-NF-*κ*B and p-IkB*α* groups. Compared with M group, # and * represent *p* < 0.05, ## and ** represent *p* < 0.01, ### and *** represents *p* < 0.001, #### and **** means *p* < 0.0001.

To examine the effect of the branches and leaves of *S. indicus* on the NF-*κ*B pathway, the right ears of the HMBL group, which exhibited the most effective anti-inflammatory results in the preceding experiment, were selected, alongside the left and right ears of the M group, to determine the levels of NF-*κ*B/*p*65, *p*-NF-κB/*p*65, IkB*α*, *p*-IkB*α* (32 + 36 sites) by Western blot. As shown in Figure 5, the HMBL group showed a significant reduction in protein levels of NF-*κ*B, *p*-NF-*κ*B, IkB*α*, and *p*-IkB*α* in comparison to the M group (*p* < 0.01). These findings demonstrate that in a mouse ear swelling model induced by xylene, the methanolic extract of branches and leaves has an anti-inflammatory effect on inflammatory ear tissue cells by inhibiting the NF-κB signaling pathway.

### 3.7 Cytotoxicity assay

**FIGURE 6:**
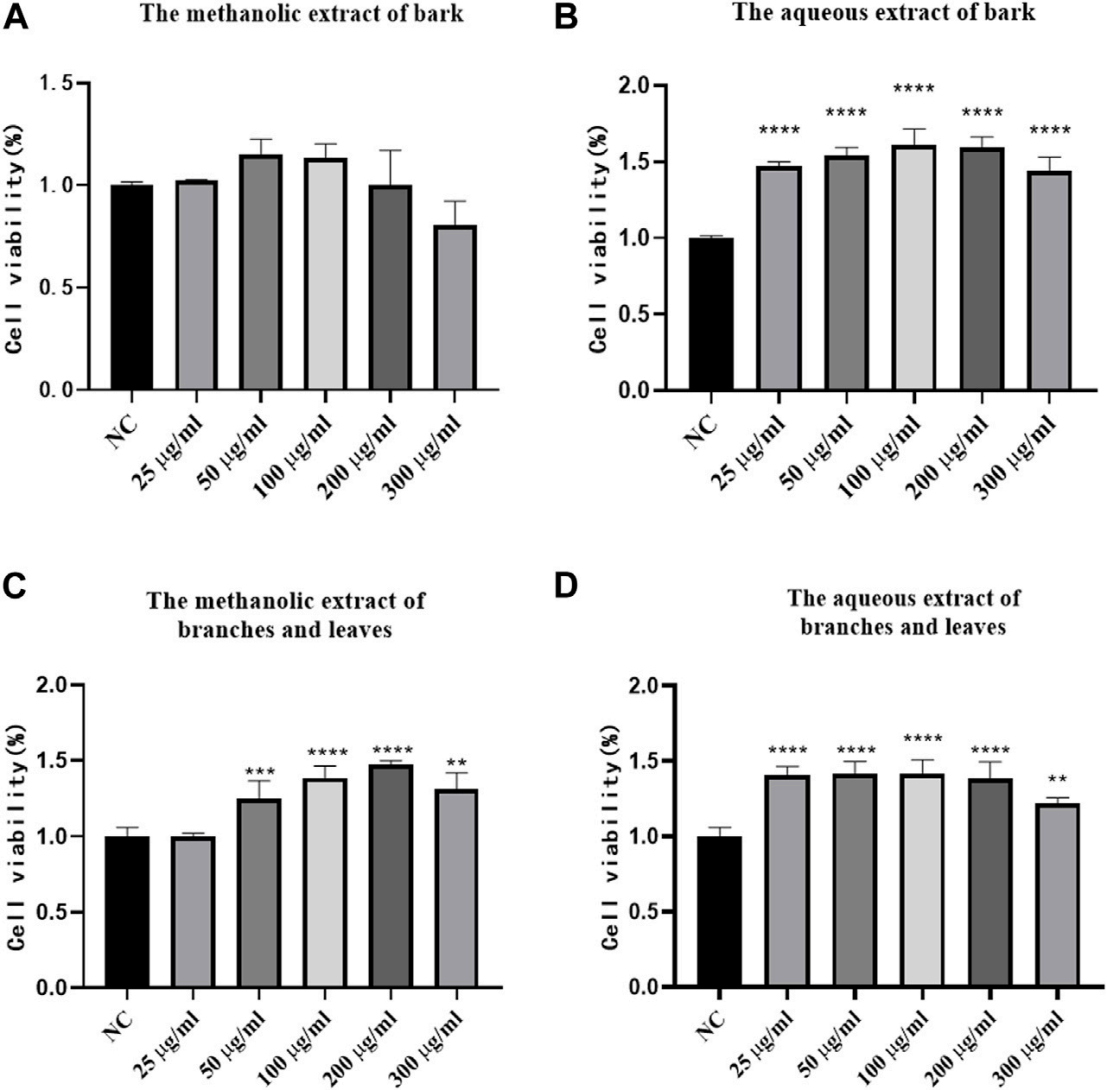
The results of Cytotoxicity test. **(A)** Cytotoxicity test results of different dosages of bark methanolic extract group. **(B)** Cytotoxicity test results of different dosages of bark aqueous extract group. **(C)** Cytotoxicity test results of different dosages of branches and leaves methanolic extract group. **(D)** Cytotoxicity test results of different dosages of branches and leaves aqueous extract group. NC was blank control group. *p < 0.05, **p < 0.01, ***p < 0.001, ****p < 0.0001 compared with model group.

To determine the cytotoxicity of *S. indicus* metabolites, the CCK-8 method was employed. According to Figure 6, cytotoxicity was not detected at methanolic metabolites dosage levels below 200 μg/mL for the bark (Figure 6A). The aqueous metabolites of the bark, and the methanolic and aqueous metabolites of branches and leaves had no cytotoxicity below 300 μg/mL (Figures 6B–D). The results showed that the metabolites of bark, branches and leaves of *S. indicus* possess a low toxicity advantage.

## 4 Discussion

In this study, a thorough investigation into the bark, branches, and leaves of *S. indicus* was carried out using various biological methods. The study showed that the bark, branches and leaves of *S. indicus* possess analgesic and anti-inflammatory properties. Additionally, the branches and leaves of *S. indicus* exhibit anti-inflammatory and analgesic effects similar to those of their bark. These activities are linked to the reduction of TNF-*α*, IL-1*β*, and IL-6 levels and the elevation of IL-4 and IL-10 levels. The anti-inflammatory effect of branches and leaves of *S. indicus* is related to the NF-*κ*B pathway. Furthermore, all *S. indicus* metabolites exhibit low toxicity levels.

The classical analgesic evaluation models (acetic acid writhing test and hot plate test) were used to investigate the analgesic activity of the bark, branches and leaves of *S. indicus*. The acetic acid writhing test induces nociceptive sensation in the primary afferent through the excessive production of inflammatory mediators in peripheral tissues leading to a writhing response in mice (Li et al., 2020; Katole et al., 2022). It is commonly used as a model to assess peripheral analgesia. In the hot plate test, mice are placed on a hot plate at a fixed temperature, and a licking paw response is observed that requires central nervous system coordination after a period of time, so this model is often used to assess the effects of potent central analgesics (Wang et al., 2019). In this study, different metabolites of the bark, branches and leaves of *S. indicus* had central and peripheral analgesic effects. When comparing the analgesic activity of the bark, branches and leaves in the same extraction solvent and at the same dose, there was no significant difference between them in the number of writhing and pain threshold indices in the mice, indicating that there was no significant difference in their analgesic effects and that they were similar in analgesic action.

To evaluate the anti-inflammatory activity of different metabolites of *S. indicus* bark, branches and leaves, xylene-induced edema assays were performed. Since xylenes are reported to cause acute edema by triggering the release of inflammatory mediators, the animal model of inflammation induced by xylenes, a widely used animal model, can be used for preliminary screening of potential anti-inflammatory drugs (Dong et al., 2017). The xylene-induced ear swelling test results indicated a significant reduction in the degree of ear edema when treated with various extracts from the bark, branches, and leaves of *S. indicus*. A comparison of the anti-inflammatory activity among these metabolites revealed no significant difference in their capacity to inhibit ear swelling at the same dose, indicating their similar activity.

Cytokines typically have pro- and anti-inflammatory effects, and their balance determines the direction of the inflammatory response (Opal and DePalo, 2000). In this study, we evaluated the anti-inflammatory efficacy by assessing the levels of three pro-inflammatory factors (TNF-*α*, IL-1*β*, and IL-6) and two anti-inflammatory factors (IL-4 and IL-10) present in the local ear tissue of mice. The pro-inflammatory cytokines IL-1*β*, IL-6, and TNF-*α* are involved in the early and ongoing inflammatory response. TNF-*α* is a master regulator of inflammation, and IL-1*β* is the central medium of the immune and inflammatory reaction, capable of promoting the production of downstream pro-inflammatory cytokines such as IL-6 and COX-2 (Standiford, 2000). In the current investigation, the different extracts of bark, branches and leaves of *S. indicus* could inhibit inflammation by decreasing the levels of IL-1*β*, IL-6, and TNF-*α* in the early stage of inflammation. Among the anti-inflammatory cytokines, IL-4 and IL-10, secreted by Th2 cells, are effector cytokines with anti-inflammatory activity (Salazar-Montes et al., 2000). IL-4 mediates humoral immunity and can inhibit the production of TNF-*α* and IL-6 in monocytes (Standiford, 2000). Regarding the inflammatory response, IL-10 can reduce antigen presentation by downregulating the expression of major histocompatibility antigen II (MHC II) on the monocyte surface and inhibiting the activation, migration, and adhesion of inflammatory cells (Brown et al., 2000). The different metabolites from the bark, branches and leaves of *S. indicus* were found to inhibit the pro-inflammatory factors produced in the early stage of inflammation and the further development of inflammation by increasing the levels of IL-4 and IL-10. By inhibiting TNF-*α*, IL-1*β*, and IL-6 while promoting the level of IL-4 and IL-10, the bark, branches and leaves of *S. indicus* inhibit inflammatory responses. The effects of the bark, branches and leaves on inflammatory factors are similar.

To intuitively reflect and explore the anti-inflammatory effect and analgesic activities of the branches and leaves from *S. indicus*, we conducted histological examination analysis and Western blot. Histological studies showed that the branches and leaves of *S. indicus* could reduce the thickness of the outer ear membrane and restore the arrangement of some membrane structures in the ears of mice with inflammation, which had a significant swelling effect. NF-*κ*B is a regulatory signal involved in the transcription of inflammatory cytokine genes and plays a central role in inflammation and general immune responses. Normally, almost all NF-*κ*B is cytoplasmically bound in an inactive form, inhibitor κB (IκB), but upon stimulation, IκB is rapidly degraded, and activated NF-*κ*B is released into the nucleus (Leslie et al., 2000; Lawrence, 2009; Wang et al., 2023). It was tentatively suggested that the anti-inflammatory and analgesic activities of *S. indicus* branches and leaves were related to the NF-*κ*B pathway by measuring the expression levels of NF-*κ*B/p65, *p*-NF-*κ*B/p65, IkB*α*, and *p*-IkB*α* in ear tissues. Compared with the NC group, the protein levels of NF-*κ*B, *p*-NF-*κ*B, IkB*α*, and *p*-IkB*α* were significantly increased in the M group, indicating that the NF-*κ*B pathway was successfully activated. The levels of NF-*κ*B, *p*-NF-*κ*B, IkB*α,* and *p*-IkB*α* proteins showed significant reduction with the high-dosage methanolic metabolites of branches and leaves, which suggests inhibition of NF-*κ*B pathway. The results of the inflammatory factors and pathway proteins were analyzed. It has been speculated that the analgesic and anti-inflammatory effects of the branches and leaves of *S. indicus* are caused by inhibiting the levels of pro-inflammatory factors such as TNF-*α*, IL-1*β*, and IL-6, which could activate the NF-*κ*B pathway inflammatory response, and by increasing the levels of anti-inflammatory factors such as IL-4 and IL-10 to inhibit the development of inflammation.

*S. Indicus*, belonging to the *Streblus* genus, has received limited research attention both domestically and internationally. The genus *Streblus* Lour. (Moraceae) comprises 22 plant species found in East and Southeast Asia (Zhang et al., 2021). Research on this genus has focused on *Streblus ilicifolius*, *Streblus asper*, and *Streblus indicus*. These species have a lengthy history of use in traditional Chinese medicine. *S. asper* and *S. indicus* have been reported to have analgesic effects (He, 2012; Chamariya et al., 2022). *S. ilicifolius*, *S. asper*, and *S. indicus* have anti-inflammatory effects. Studies have found that *S. ilicifolius* contains numerous compounds with anti-inflammatory effects and two compounds were found to significantly inhibit the expression of COX-2, iNOS, and NF-*κ*B/p65 (Huang et al., 2021; Huang et al., 2023). One study discovered that the ethanol extract of *S. asper* significantly inhibited LPS-induced COX-2 expression and iNOS mRNA (Chamariya et al., 2022). Studies have shown that coumarins such as scopoletin and umbelliferone from *S. indicus* can exert anti-inflammatory and analgesic effects by regulating the NF-*κ*B pathway (Wu et al., 2021; Fan et al., 2022). It is consistent with the results of the present study and provides a direction for the follow-up study of *S. indicus*.

The present study is only a preliminary investigation of the analgesic and anti-inflammatory effects of the bark, branches and leaves of *S. indicus*. Further evaluation of the analgesic and anti-inflammatory-related cytokine levels and signaling pathways should be conducted. Based on the current studies on the bark, branches and leaves of *S. indicus*, future research will subsequently investigate the exact mechanisms of analgesic and anti-inflammatory effects of these extracts. Then try to extract as many compounds from them as possible with analgesic and anti-inflammatory effects.

## 5 Conclusion

This paper explains the scientific basis of ethnic medicinal substitution by studying the pharmacological activity of *S. indicus*. The results also provide theoretical support for the rational development and utilization of the branches and leaves of *S. indicus*.

## Data Availability

The original contributions presented in the study are included in the article/Supplementary Material, further inquiries can be directed to the corresponding authors.

## References

[B1] Agrawal R. Sandhu S. K. Sharma I. Kaur I. P. (2015). Development and evaluation of curcumin-loaded elastic vesicles as an effective topical anti-inflammatory formulation. AAPS PharmSciTech 16 (2), 364–374. 10.1208/s12249-014-0232-6 25319056 PMC4370954

[B2] Alam F. Hanif M. Rehman A. U. Ali S. Jan S. (2022). *In vitro*, *in vivo* and *in silico* evaluation of analgesic, anti-inflammatory, and anti-pyretic activity of salicylate rich fraction from *Gaultheria trichophylla* Royle (Ericaceae). J. Ethnopharmacol. 301, 115828. 10.1016/j.jep.2022.115828 36240979

[B3] Arulselvan P. Fard M. T. Tan W. S. Gothai S. Fakurazi S. Norhaizan M. E. (2016). Role of antioxidants and natural products in inflammation. Oxid. Med. Cell. Longev. 2016, 5276130. 10.1155/2016/5276130 27803762 PMC5075620

[B4] Arya N. Prakash O. Vivekanand P. A. K. (2016). Central and peripheral analgesic activity of turmeric rhizome collected from uttarakhand, India. Asian J. Org. Chem. 28 (5), 1024–1026. 10.14233/ajchem.2016.19571

[B5] Ballester P. Cerdá B. Arcusa R. Marhuenda J. Yamedjeu K. Zafrilla P. (2022). Effect of ginger on inflammatory diseases. Molecules 27 (21), 7223. 10.3390/molecules27217223 36364048 PMC9654013

[B6] Brown N. L. Alvi S. A. Elder M. G. Bennett P. R. Sullivan M. H. (2000). The regulation of prostaglandin output from term intact fetal membranes by anti-inflammatory cytokines. Immunology 99 (1), 124–133. 10.1046/j.1365-2567.2000.00942.x 10651950 PMC2327135

[B7] Chamariya R. Raheja R. Suvarna V. Bhandare R. (2022). A critical review on phytopharmacology, spectral and computational analysis of phytoconstituents from *Streblus asper* Lour. Phytomedicine Plus 2 (1), 100177. 10.1016/J.PHYPLU.2021.100177

[B8] Chen Y. H. Luo R. Lei S. S. Li B. Zhou F. C. Wang H. Y. (2020). Anti-inflammatory effect of Ganluyin, a Chinese classic prescription, in chronic pharyngitis rat model. BMC Complement. Med. Ther. 20 (1), 265. 10.1186/s12906-020-03057-5 32859182 PMC7456022

[B9] De Meij J. Alfanek Z. Morel L. Decoeur F. Leyrolle Q. Picard K. (2021). Microglial cannabinoid type 1 receptor regulates brain inflammation in a sex-specific manner. Cannabis Cannabinoid Res. 6 (6), 488–507. 10.1089/can.2020.0170 34591647 PMC8713265

[B10] Dong D. Zhou N. N. Liu R. X. Xiong J. W. Pan H. Sun S. Q. (2017). Sarsasapogenin-AA13 inhibits LPS-induced inflammatory responses in macrophage cells *in vitro* and relieves dimethylbenzene-induced ear edema in mice. Acta Pharmacol. sin. 38 (5), 699–709. 10.1038/aps.2016.180 28239159 PMC5457694

[B11] Duan Y. Ying Z. M. He F. Ying X. X. Jia L. Q. Yang G. L. (2021). A new skeleton flavonoid and a new lignan from *Portulaca oleracea* L. and their activities. Fitoterapia 153, 104993. 10.1016/J.FITOTE.2021.104993 34284073

[B12] Elkahloun A. G. Rodriguez Y. Alaiyed S. Wenzel E. Saavedra J. M. (2019). Telmisartan protects a microglia cell line from LPS injury beyond AT1 receptor blockade or PPARγ activation. Mol. Neurobiol. 56 (5), 3193–3210. 10.1007/s12035-018-1300-9 30105672 PMC6677563

[B13] Fan Z. X. Tang D. Wu Q. Huang Q. Song J. Long Q. P. (2022). Scopoletin inhibits PDGF-BB-induced proliferation and migration of airway smooth muscle cells by regulating NF-κB signaling pathway. Allergol. Immunopathol. 50 (1), 92–98. 10.15586/aei.v50i1.517 34965643

[B14] Garateix A. Salceda E. Menéndez R. Regalado E. L. López O. García T. (2011). Antinociception produced by Thalassia testudinum extract BM-21 is mediated by the inhibition of acid sensing ionic channels by the phenolic compound thalassiolin B. Mol. Pain 7 (1), 10. 10.1186/1744-8069-7-10 21261973 PMC3037906

[B15] Gugliandolo E. Macrì F. Fusco R. Siracusa R. D'Amico R. Cordaro M. (2021). The protective effect of snail secretion filtrate in an experimental model of excisional wounds in mice. Vet. Sci. 8 (8), 167. 10.3390/vetsci8080167 34437489 PMC8402640

[B16] Han J. Song J. G. (2008). Chronopharmacological study on analgesia and anti-inflammatory of ji-ming-san. *Chin. J. Exp. Traditional Med. Formula*e 09, 63–67. 10.13422/j.cnki.syfjx.2008.09.026

[B17] He R. J. (2012). Constituents of the bark of Streblus indicus and their biological activitise. PhD thesis. China (Guangxi): Guangxi Normal University.

[B18] Henein M. Y. Vancheri S. Longo G. Vancheri F. (2022). The role of inflammation in cardiovascular disease. Int. J. Mol. Sci. 23 (21), 12906. 10.3390/ijms232112906 36361701 PMC9658900

[B19] Hu Z. H. Kong Y. Y. Ren J. J. Huang T. J. Wang Y. Q. Liu L. X. (2020). Kidney and lung tissue modifications after BDL-induced liver injury in mice are associated with increased expression of IGFBPrP1 and activation of the NF-κB inflammation pathway. Int. J. Clin. Exp. Pathol. 13 (2), 192–202. 32211099 PMC7061808

[B20] Huang C. Dong C. Zhu Y. Yu Y. Jin H. Zhang Y. (2021a). Duhaldea pterocaula (franch.) anderb. Attenuates nociception and inflammation via GABAA receptors. Front. Pharmacol. 12, 753128. 10.3389/fphar.2021.753128 34795587 PMC8592923

[B21] Huang Y. Huang X. S. Tian G. B. Zhang W. X. Su S. S. Xu X. (2021b). Two new amide glycosides with anti-inflammatory activity from the leaves of *Streblus ilicifolius* (Vidal) Corner. Nat. Prod. Res. 36 (6), 1485–1493. 10.1080/14786419.2021.1893318 33673782

[B22] Huang Y. Pan L. W. Chang Y. L. Liang X. Q. Hou P. Ren C. Y. (2023). Megastigmane glycosides from *Streblus ilicifolius* (S.Vidal) Corner and their anti-inflammatory activity. Phytochemistry 208, 113606. 10.1016/J.PHYTOCHEM.2023.113606 36736939

[B23] Jain A. P. Bhandarkar S. Rai G. Yadav A. K. Lodhi S. (2016). Evaluation of parmotrema reticulatum taylor for antibacterial and antiinflammatory activities. Indian J. Pharm. Sci. 78 (1), 94–102. 10.4103/0250-474x.180241 27168687 PMC4852582

[B24] Jiang J. Yan L. Shi Z. Wang L. Shan L. Efferth T. (2019). Hepatoprotective and anti-inflammatory effects of total flavonoids of Qu Zhi Ke (peel of Citrus changshan-huyou) on non-alcoholic fatty liver disease in rats via modulation of NF-κB and MAPKs. Phytomedicine 64, 153082. 10.1016/j.phymed.2019.153082 31541796

[B25] Katole N. T. Kale J. S. Salankar H. V. (2022). Evaluation of the antinociceptive action of simvastatin in mice. Cureus J med Sci. 14 (7), e26910. 10.7759/cureus.26910 PMC9376206 35983393

[B26] Lawrence T. (2009). The nuclear factor NF-kappaB pathway in inflammation. *Cold Spring Harb. Perspect. Bio*l. 1 (6), a001651. 10.1101/cshperspect.a001651 20457564 PMC2882124

[B27] Leslie K. K. Lee S. L. Woodcock S. M. Davies J. K. McDuffie R. S. Hirsch E. (2000). Acute intrauterine infection results in an imbalance between pro- and anti-inflammatory cytokines in the pregnant rabbit. Am. J. Reprod. Immunol. 43 (5), 305–311. 10.1111/j.8755-8920.2000.430510.x 10872611

[B28] Li F. Huo J. Zhuang Y. Xiao H. Wang W. Huang L. (2020). Anti-nociceptive and anti-inflammatory effects of the ethanol extract of *Arenga pinnata* (Wurmb) Merr. fruit. J. Ethnopharmacol. 248, 112349. 10.1016/j.jep.2019.112349 31756450

[B29] Lin Y. F. Yi Z. Zhao Y. H. (2003). Color spectrum of medicine of Dai nationality in China (in Chinese). China: Yunnan Nationalities Publishing House, 582.

[B30] Mahmoud A. M. Desouky E. M. Hozayen W. G. Bin-Jumah M. El-Nahass E. S. Soliman H. A. (2019). Mesoporous silica nanoparticles trigger liver and kidney injury and fibrosis via altering TLR4/NF-κB, JAK2/STAT3 and Nrf2/HO-1 signaling in rats. Biomolecules 9 (10), 528. 10.3390/biom9100528 31557909 PMC6843412

[B31] Maria I. (2021). Inflammation induces zebrafish regeneration. Neural Regen. Res. 16 (9), 1693–1701. 10.4103/1673-5374.306059 33510057 PMC8328752

[B32] Murakami Y. Kawata A. Suzuki S. Fujisawa S. (2018). Cytotoxicity and pro-/anti-inflammatory properties of cinnamates, acrylates and methacrylates against RAW264.7 cells. Vivo 32 (6), 1309–1322. 10.21873/invivo.11381 PMC6365727 30348683

[B33] Opal S. M. DePalo V. A. (2000). Anti-inflammatory cytokines. Chest 117 (4), 1162–1172. 10.1378/chest.117.4.1162 10767254

[B34] Ramírez W. Torralba D. Bourg V. Lastre M. Perez O. Jacquet A. (2022). Immunogenicity of a novel anti-allergic vaccine based on house dust mite purified allergens and a combination adjuvant in a murine prophylactic model. Front. Allergy 3, 1040076. 10.3389/FALGY.2022.1040076 36479436 PMC9720566

[B35] Salazar-Montes A. Delgado-Rizo V. Armendáriz-Borunda J. (2000). Differential gene expression of pro-inflammatory and anti-inflammatory cytokines in acute and chronic liver injury. Hepatol. Res. 16 (3), 181–194. 10.1016/S1386-6346(99)00048-0

[B36] Shaik-Dasthagirisaheb Y. B. Varvara G. Murmura G. Saggini A. Caraffa A. Antinolfi P. (2013). Role of vitamins D, E and C in immunity and inflammation. J. Biol. Regul. Homeost. Agents 27 (2), 291–295. 10.1139/cjpp-2013-0116 23830380

[B37] Sophie B. Sophie N. Elise B. Catherine P. (2022). Early protective role of inflammation in cardiac remodeling and heart failure: Focus on TNFα and resident macrophages. Cells 11 (7), 1249. 10.3390/cells11071249 35406812 PMC8998130

[B38] Standiford T. J. (2000). Anti-inflammatory cytokines and cytokine antagonists. Curr. Pharm. Des. 6 (6), 633–649. 10.2174/1381612003400533 10788601

[B39] Wang S. S. Zhou S. Y. Xie X. Y. Zhao L. Fu Y. Cai G. Z. (2020). Comparison of the acute toxicity, analgesic and anti-inflammatory activities and chemical composition changes in Rhizoma anemones Raddeanae caused by vinegar processing. Bmc Complement. Med. 20 (1), 7. 10.1186/s12906-019-2785-0 PMC7076870 32020868

[B40] Wang X. F. Wang D. R. Deng B. Y. Yan L. T. (2023). Syringaresinol attenuates osteoarthritis via regulating the NF-κB pathway. Int. Immunopharmacol. 118, 109982. 10.1016/j.intimp.2023.109982 36989902

[B41] Wang Y. Zhao T. T. Dai Y. L. Yang R. C. Li S. J. (2019). Study on analgesic and sedative effects of Tianmu Jiangya Tablets in mouse model. Chin. J. Integr. Med. Cardio-Cerebrovascular Dis. 17 (3), 364–367. 10.12102/j.issn.1672-1349.2019.03.011

[B42] Wu G. F. Nie W. B. Wang Q. Hao Y. G. Gong S. H. Zheng Y. X. (2021). Umbelliferone ameliorates complete freund adjuvant–induced arthritis via reduction of NF-κB signaling pathway in osteoclast differentiation. Inflammation 44 (4), 1315–1329. 10.1007/s10753-021-01418-x 33484396

[B43] Xue D. Zhou X. Qiu J. (2021). Cytotoxicity mechanisms of plumbagin in drug-resistant tongue squamous cell carcinoma. J. Pharm. Pharmacol. 73 (1), 98–109. 10.1093/JPP/RGAA027 33791802

[B44] Yunnan Food and Drug Administration (2013). Yunnan provincial standard of Chinese medicinal materials (Volume7, 2005 edition)”. China, Kunming: Yunnan Science and Technology Press, 73.

[B45] Zeng X. Z. He L. G. Wang S. Wang K. Zhang Y. Y. Tao L. (2016). Aconine inhibits RANKL-induced osteoclast differentiation in RAW264.7 cells by suppressing NF-κB and NFATc1 activation and DC-STAMP expression. Acta Pharmacol. Sin. 37 (2), 255–263. 10.1038/aps.2015.85 26592521 PMC4753374

[B46] Zhang G. R. Huang X. S. Huang Y. Li J. (2021b). Research progress on chemical constituents and pharmacological activities of plants from *Streblus* . Chin. Tradit. Herb. Drugs. 52 (19), 6066–6075. 10.7501/j.issn.0253-2670.2021.19.030

[B47] Zhang R. T. Wu S. Q. Ding Q. Fan Q. Z. Dai Y. Guo S. W. (2021a). Recent advances in cell membrane-camouflaged nanoparticles for inflammation therapy. Drug Deliv. 28 (1), 1109–1119. 10.1080/10717544.2021.1934188 34121563 PMC8205088

[B48] Zhuang Z. Ye G. Huang B. (2017). Kaempferol alleviates the interleukin-1β-induced inflammation in rat osteoarthritis chondrocytes via suppression of NF-κB. Med. Sci. Monit. 23, 3925–3931. 10.12659/msm.902491 28806392 PMC5566200

