# Supplementary material for: Anti-inflammatory and analgesic effects of Streblus indicus
Source: Front Pharmacol. 2023 Sep 27;14:1249234. doi: 10.3389/fphar.2023.1249234 (PMC10565225; doi:10.3389/fphar.2023.1249234)
Supplement: Supplementary file 1 [file DataSheet1.docx]

Supplementary Material

Anti-inflammatory and analgesic effects of *Streblus indicus*

Yan-Qing Xie^1,†^, Jing-Yao Huang^1,†^, Yun-Xiu Chen^1^, Qian Zhou^1^, Qi-Xiu Zhou^1^, Zhu-Ya Yang^1^, Shi-Kui Xu^2^, Wen-Hong Tan^1,^*, Lu Liu^1,^*

*** Correspondence:** Lu Liu: [todayliulu@163.com](mailto:todayliulu@163.com)

Wen-Hong Tan: [twh85087@126.com](mailto:twh85087@126.com)

†These authors contributed equally to this work and share first authorship

# Supplementary Figures


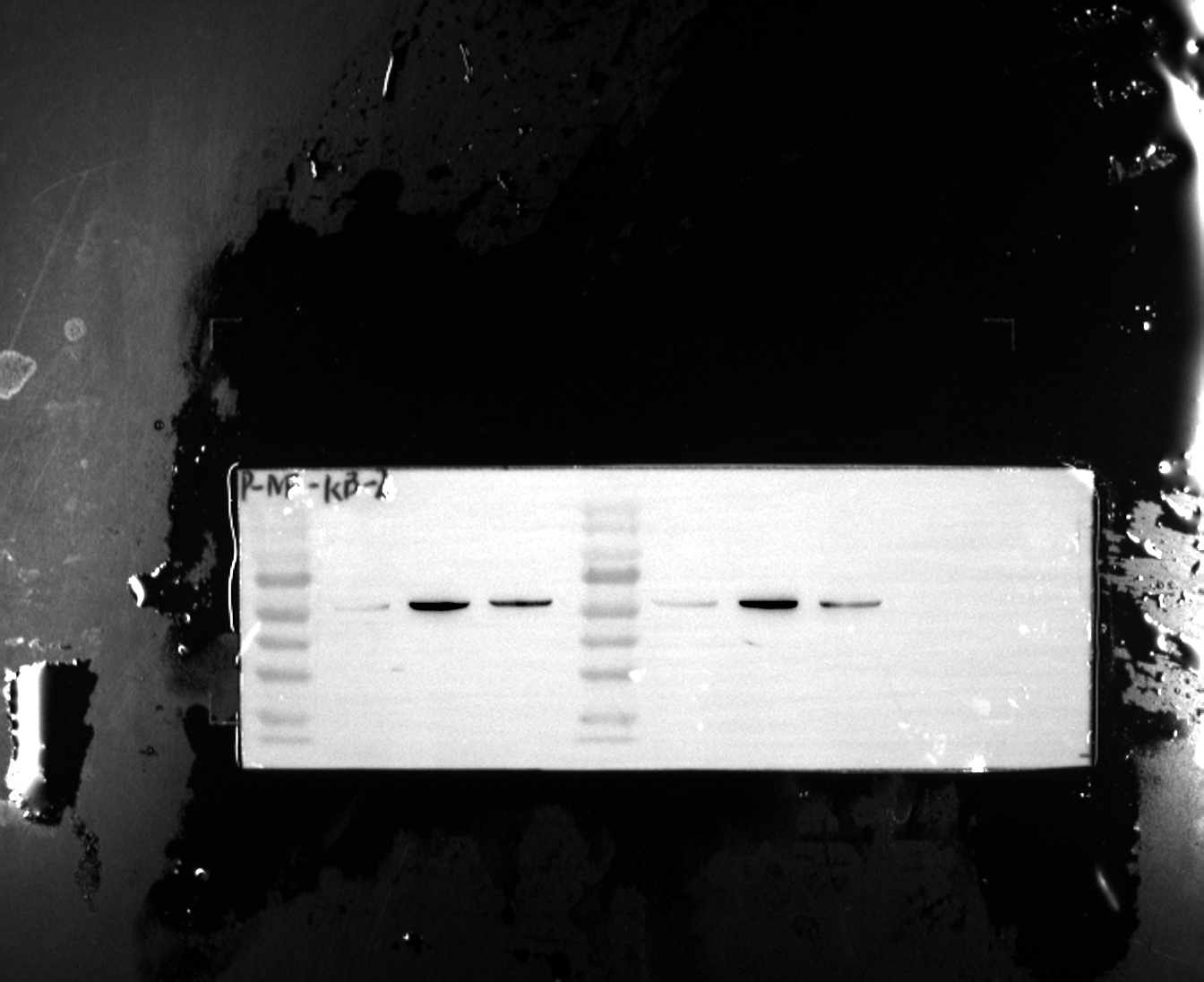


**Supplementary Figure 1.** The whole original gel of *p*-NF-*k*B of the NC, M and HMBL group. The red box shows the data used in the article.


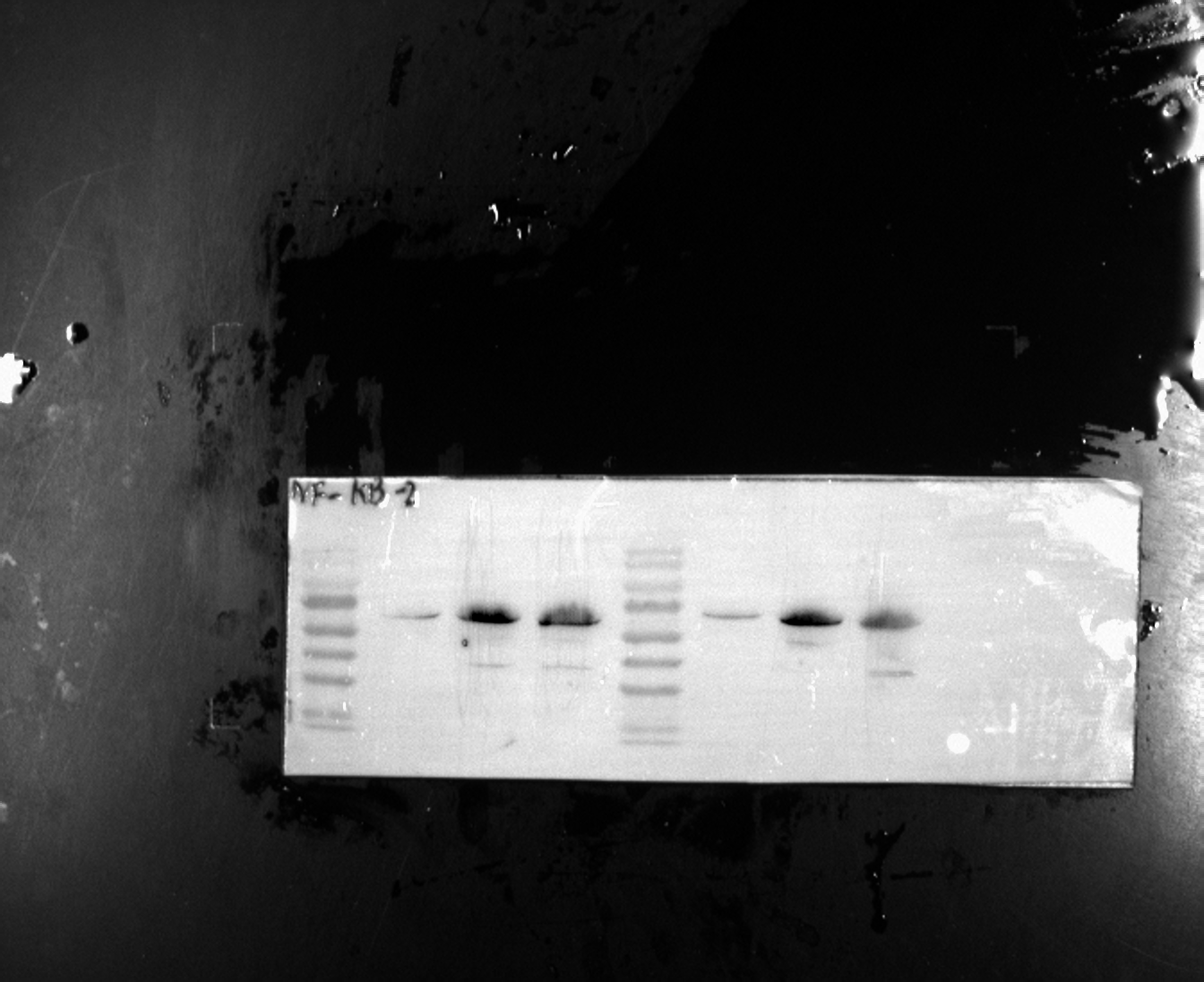


**Supplementary Figure 2.** The whole original gel of NF-*k*B of the NC, M and HMBL group. The red box shows the data used in the article.


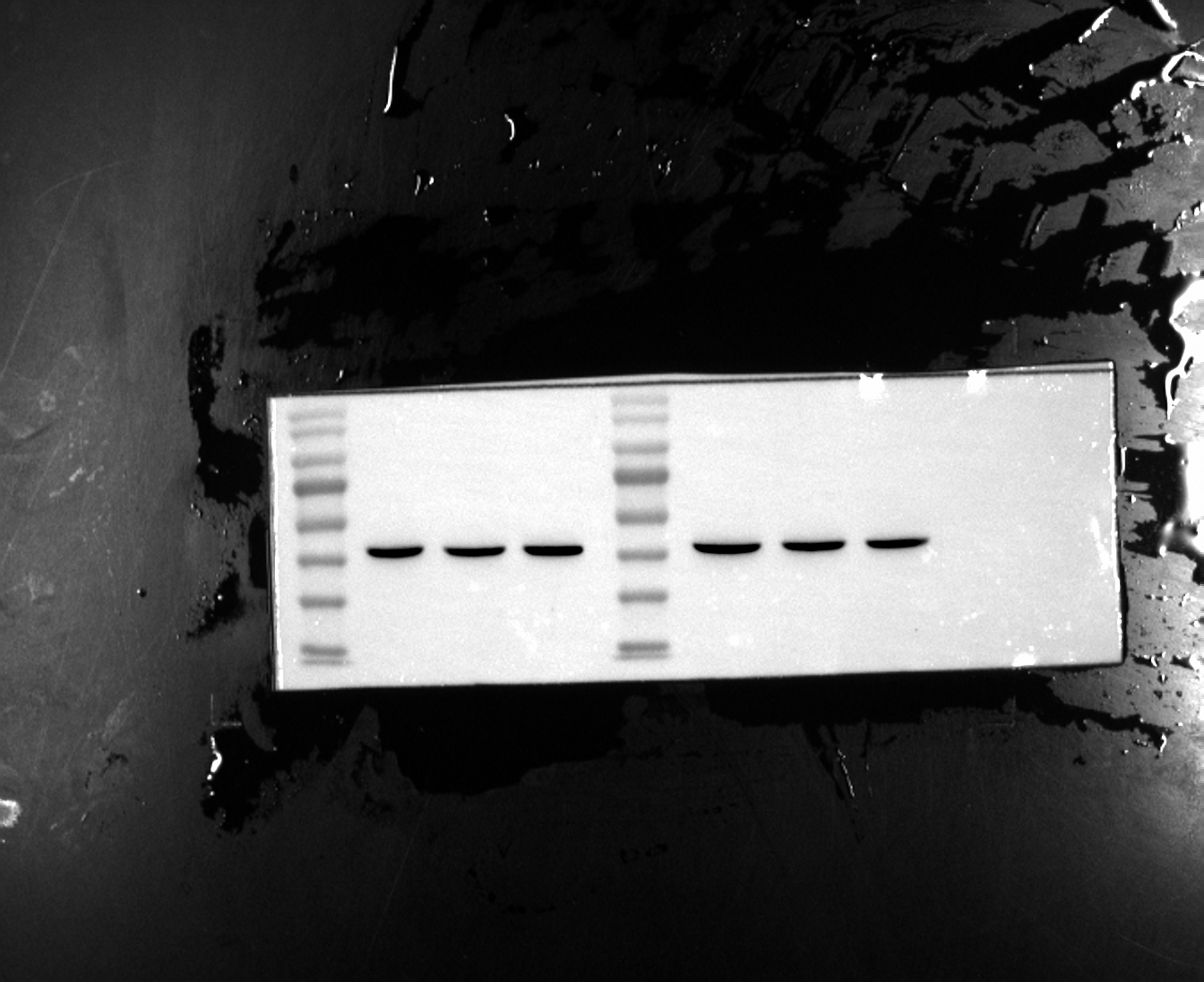


**Supplementary Figure 3.** The whole original gel of *β*-actin of the NC, M and HMBL group. The red box shows the data used in the article .


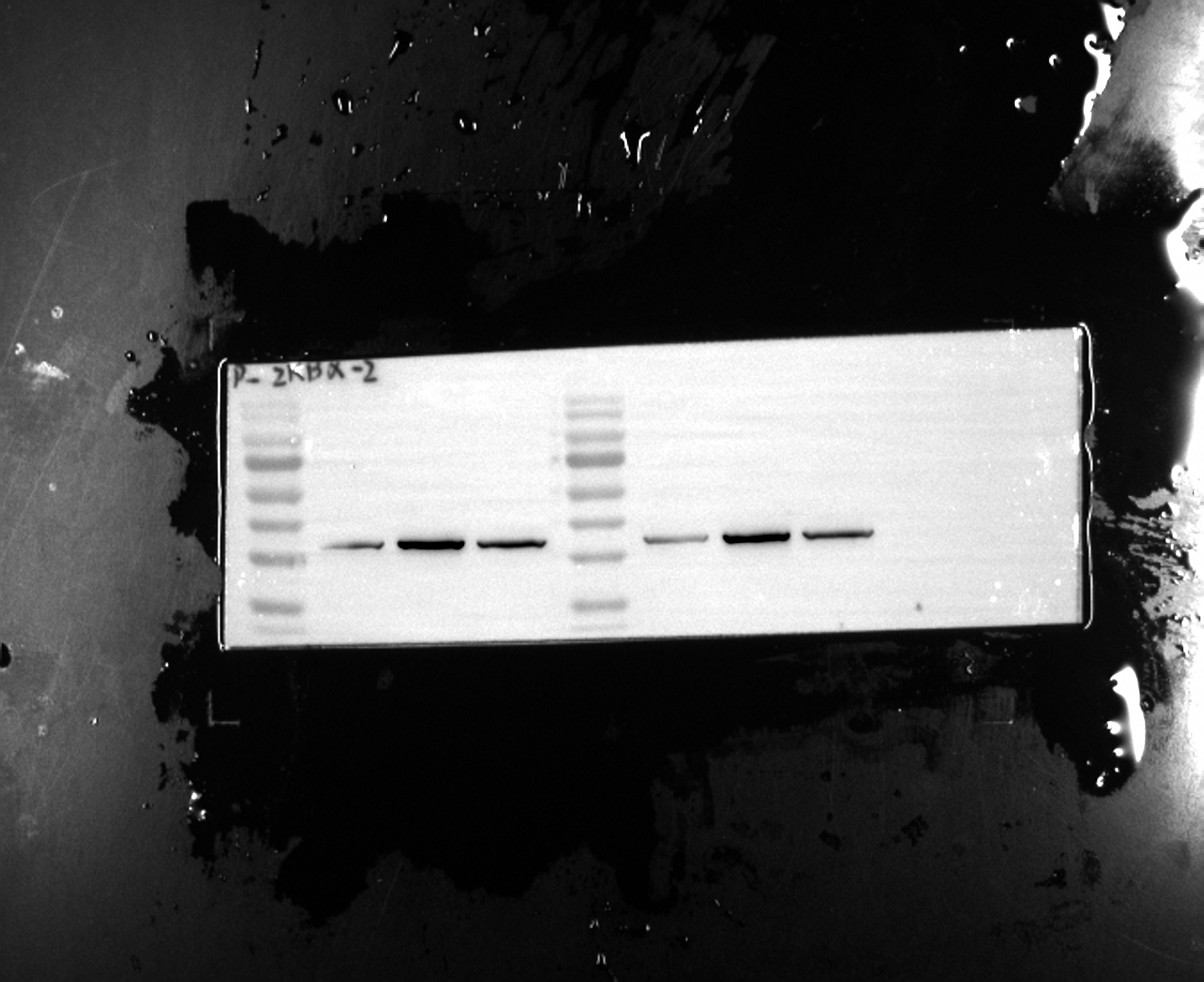


**Supplementary Figure 4.** The whole original gel of *p*-IkB*α* of the NC, M and HMBL group. The red box shows the data used in the article.


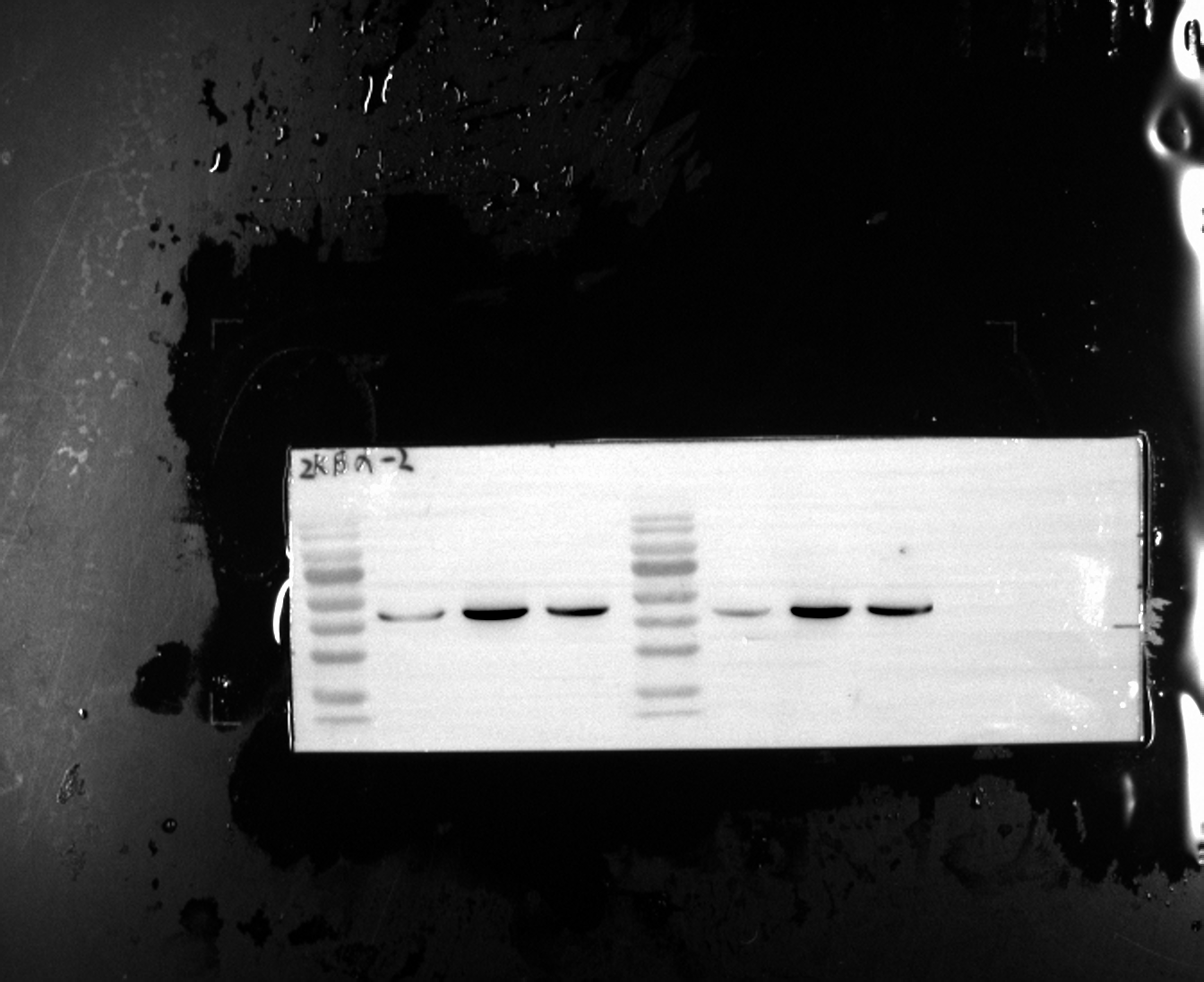


**Supplementary Figure 5.** The whole original gel of IkB*α* of the NC, M and HMBL group. The red box shows the data used in the article.


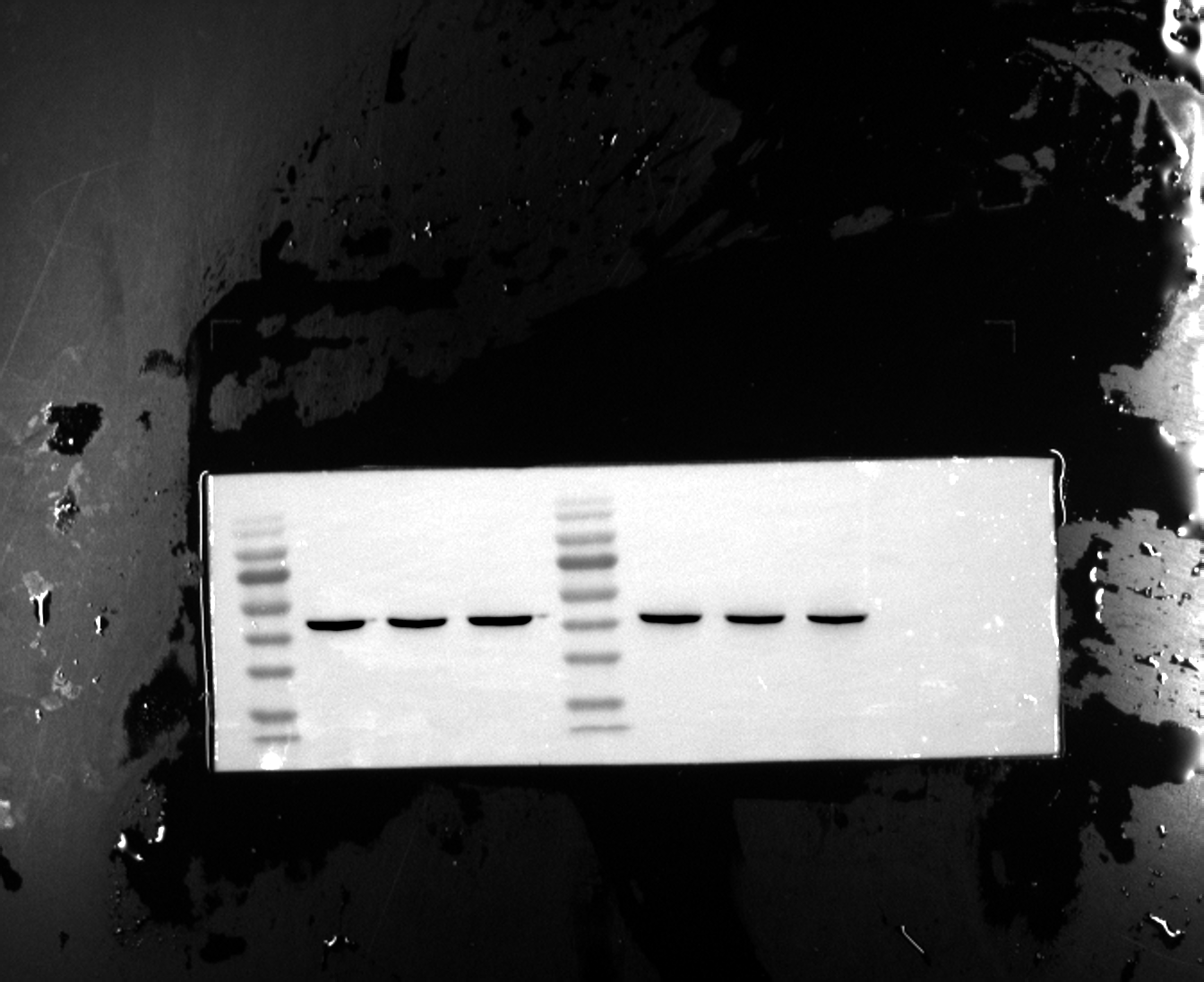


**Supplementary Figure 6.** The whole original gel of *β*-actin of the NC, M and HMBL group. The red box shows the data used in the article.

# Supplementary Tables

**Supplementary Table 1.** The results of Acetic acid writhing test (‾x±s, n=10)

| **Group** | **Dose(mg/kg)** | **Female mice**  **Number of torsion** | **Male mice**  **Number of torsion** |
| --- | --- | --- | --- |
| M | - | 37.88±7.94 | 40.00±4.86 |
| P | 150.00 | 7.750±5.90 | 11.00±8.79 |
| LAB | 5.77 | 26.89±8.16 | 26.10±7.36 |
| HAB | 23.08 | 25.83±4.45 | 23.38±9.39 |
| LABL | 5.77 | 26.50±6.07 | 24.10±6.87 |
| HABL | 23.08 | 26.00±3.42 | 37.83±9.09 |
| LMB | 46.16 | 27.33±9.14 | 28.33±5.83 |
| HMB | 184.64 | 23.38±7.15 | 26.67±5.78 |
| LMBL | 46.16 | 26.88±9.60 | 29.89±7.03 |
| HMBL | 184.64 | 24.75±5.15 | 28.67±4.46 |

**Supplementary Table 2.** The Results of Xylene-induced Ear Swelling Test (‾x±s, n=10)

| **Groups** | **Dose (mg/kg)** | **Degree of auricle swelling** | **Inhibit rate of inflammation (%)** |
| --- | --- | --- | --- |
| M | - | 11.34±4.73 | - |
| P | 10.00 | 5.88±2.17 | 48.21 |
| LAB | 5.77 | 4.58±2.33 | 59.64 |
| HAB | 23.08 | 3.64±3.01 | 67.93 |
| LABL | 5.77 | 4.22±4.21 | 62.83 |
| HABL | 23.08 | 6.36±2.92 | 43.91 |
| LMB | 46.16 | 4.87±2.22 | 57.07 |
| HMB | 184.64 | 2.66±2.39 | 76.59 |
| LMBL | 46.16 | 6.71±2.39 | 44.33 |
| HMBL | 184.64 | 6.90±3.26 | 39.17 |

**Supplementary Table 3.** Results of Inflammatory Cytokines Assay (‾x±s, n=10)


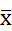


| **Gourps** | **Dose**  **(mg/kg)** | **TNF-*α* (pg/g)** | **IL-1*β* (pg/g)** | **IL-6 (pg/g)** | **IL-4 (pg/g)** | **IL-10 (pg/g)** |
| --- | --- | --- | --- | --- | --- | --- |
| M | - | 6708.21±598.26 | 1205.89±70.65 | 1271.97±112.07 | 987.45±87.32 | 1597.80±360.21 |
| P | 10.00 | 4062.52±662.25 | 682.09±122.08 | 775.32±118.75 | 1562.75±167.01 | 2855.10±276.50 |
| LAB | 5.77 | 5522.56±710.04 | 913.37±131.91 | 1098.91±117.79 | 1207.95±158.01 | 2130.43±464.48 |
| HAB | 23.08 | 4744.30±677.52 | 878.85±116.95 | 1011.49±101.54 | 1273.57±156.07 | 2356.17±238.42 |
| LABL | 5.77 | 4151.42±525.09 | 799.20±133.54 | 948.56±144.15 | 1342.21±150.00 | 2544.70±433.92 |
| HABL | 23.08 | 5145.89±597.54 | 889.44±96.47 | 1034.44±129.01 | 1239.35±103.91 | 2152.25±472.08 |
| LMB | 46.16 | 5014.32±772.01 | 936.79±102.35 | 1016.60±88.27 | 1291.61±161.81 | 2379.16±362.33 |
| HMB | 184.64 | 4205.74±751.76 | 752.26±129.07 | 916.03±93.69 | 1337.48±113.77 | 2616.22±393.19 |
| LMBL | 46.16 | 5156.38±706.13 | 842.79±128.30 | 1000.55±115.94 | 1247.38±162.18 | 2067.27±333.18 |
| HMBL | 184.64 | 4227.92±873.91 | 803.30±125.90 | 972.83±89.97 | 1424.97±202.60 | 2729.79±367.91 |
